# Optimizing the Extraction of Polyphenols from Different Edible Lichens Using Response Surface Methodology and the Determination of Their Mineral and Antibacterial Properties

**DOI:** 10.3390/foods14152562

**Published:** 2025-07-22

**Authors:** Kubra Ozkan, Hatice Bekiroglu, Nur Cebi, Fatih Bozkurt, Sevda Dere, Hilmi Ozdemir, Muhammet Arici, Salih Karasu, Osman Sagdic

**Affiliations:** 1 Department of Nutrition and Dietetics, Health Sciences Faculty, Istinye University, 34010 Istanbul, Türkiye; 2 Department of Food Engineering, Faculty of Agriculture, Sirnak University, 73300 Sirnak, Türkiye; 3 Department of Food Engineering, Faculty of Chemical and Metallurgical Engineering, Yildiz Technical University, 34220 Istanbul, Türkiye; 4 Marmara Forestry Research Institute Directorate, 34457 Istanbul, Türkiye

**Keywords:** ultrasound-assisted extraction, optimization, phenolic profile, volatile compound, mineral content

## Abstract

This study employed response surface methodology for the first-time optimization of the ultrasound-assisted extraction (UAE) of the total phenolic content (TPC) and ABTS from edible lichens, including *Evernia divaricata*, *Evernia prunastri*, *Pseudevernia furfuracea*, *Bryoria fuscescens*, and *Lobaria pulmonaria*. Fourteen experimental points were generated using Design Expert Software, with the extraction temperature (25–40 °C), extraction time (5–20 min), and ethanol concentration (0–80%) as independent variables, and TPC and ABTS as dependent variables. The phenolic profile and mineral and antibacterial properties of the optimized lichen extracts were determined. Evernic and usnic acid were found in *Evernia* species. Atranorin was detected only in *P. furfuracea*. Fumarprotocetraric acid was found exclusively in *B. fuscescens* and was not detected in any of the other lichens. Calcium was found to have the highest mineral content in all the lichens, followed by potassium. *L. pulmonaria*, showing the lowest inhibition effect against all tested bacteria, while *E. divaricata* exhibited the most effective inhibition.

## 1. Introduction

Lichens are intricate associations of a symbiotic relationship between fungi (mycobionts) and one or more algae or cyanobacteria (photobionts). There are over 17,000 species of lichens around the globe, and they are pioneer species because of their outstanding capacity for tolerance to a wide range of harsh environmental circumstances. No less than 800 lichen-based products are used in the food industry, as well as perfume with further uses as dyes, pollution bioindicators, and drugs [1]. According to Culberson and Elix [2], lichen compounds are classified based on their biosynthetic origins and chemical structures. These compounds are synthesized through mevalonate, acetyl-malonate, and shikimate pathways, fundamental routes for natural metabolism in all organisms. The acetyl-malonate pathway is responsible for the biosynthesis of lichen depsides, dibenzofurans, depsidones, xanthones, anthraquinones, and chromones. Most bioactive compounds are synthesized via this pathway, with coenzyme A serving as the precursor and polyketide synthase (PKS) as the key enzyme [3]. The numerous biological characteristics of lichens’ metabolites have been identified, and they are well recognized for their therapeutic effects [4,5,6,7]. The synergistic interaction of algae and fungi in lichens results in the formation of several chemicals, known as lichen acids or secondary metabolites, which are the source of the therapeutic actions of lichens. Aliphatic, aromatic, cycloaliphatic, and terpenic compounds make up the secondary metabolites found in lichens. These compounds have important biological and pharmacological properties, including antioxidant, cytotoxic, anti-inflammatory, antiviral, and antibacterial properties [8].

Their low fat content, good protein sources, high carbohydrate content, crude fiber content, and plentiful mineral components are all thought to contribute to lichens’ nutritional value [9]. Only a few edible lichen species have been documented, and research on edible lichens is currently limited [10]. These edible lichens, which are very rich sources of nutritional properties, have been used as folk foods and traditional medicines for many years in countries such as many European countries, China, Japan, and India [11]. *Pseudevernia furfuracea* is a recognized foliose lichen utilized commercially as a preservative in food and herbal formulations, as well as in blends of spices and culinary dishes such as curries [12]. This lichen is also used to treat asthma, hypertension, and congestion in the lungs [13]. In India, the Middle East, and Niger, *Platismatia glauca* has been used as a spice or food flavor enhancer [10]. In addition, some lichens such as *Usnea florida* and *Evernia prunastri* are the choice of attention for use in food ingredients and food colorings [14]. According to reports, lichens are a healthy food source, due to their phenolic content, volatile fraction, polysaccharides, and dietetics [15]. Research focused on the identification of the naturally occurring components responsible for the bioactivity of lichens has been driven by their ethnopharmacological importance and use as a functional food. Furthermore, it is well known that the methods of extraction utilized to isolate these substances are crucial for establishing the extraction yield and the antioxidant potential of the extracts [16,17]. Common lichen compounds synthesized through the acetyl-malonate pathway include atranorin, evernic acid, thamnolic acid, umbilicaric acid, protocetraric acid, fumarprotocetraric acid, stictic acid, usnic acid, lecanoric acid, gyrophoric acid, lepraric acid, and thiophanic acid [18,19,20,21,22]. Some pathogenic bacteria have been stated to be sensitive to lichen extracts or their active substances, such as *Staphylococcus aureus*, *Escherichia coli, Proteus vulgaris*, *Enterococcus faecalis*, *Pseudomonas aeruginosa*, *Enterobacter cloacae*, *Proteus mirabilis*, *Klebsiella pneumoniae*, *Helicobacter pylori*, and *Aeromonas hydrophila* [23,24]. A different study revealed that the powerful secondary metabolite physciosporin, which is present in several lichen species from the genus Pseudocyphellaria, has anticancer potential through stifling the development and movement of colorectal cancer cells via unique methods [25].

Today, various extraction techniques are employed to create phenolic-rich plant extracts. In a nutshell, new techniques include accelerated solvents, microwave-assisted, ultrasound-assisted, supercritical fluids, boiling, and refluxing extractions [26]. When compared to traditional extraction procedures, the ultrasound-assisted extraction (UAE) methodology for extracting bioactive chemicals has advantages in terms of efficiency, energy use, and solvent use. These significant benefits have made the UAE process well known as an environmentally friendly extraction method. Cavitation is a phenomenon that contributes to the increased effectiveness of UAE. By rupturing the cell walls, this approach allows for the improved mass transit of the cell content to the solvent [27].

Response surface methodology (RSM) has been successfully used, according to prior studies, to optimize the extraction parameters of phenolics from a wide range of different foods such as fruits and plants. RSM is a statistical methodology used to model and evaluate problems containing several variables that affect the optimal response. This method aims to determine the best combination of variables to achieve optimal production conditions. Compared to traditional optimization techniques, RSM has several advantages, such as a reduced number of experiments required to assess the influence of all factors, the ability to determine optimal variable combinations, and significant time savings. RSM has been widely used in various optimization studies to minimize time, material, cost, and reagent consumption [28]. The extraction conditions for phenolics from *Usnea longissima* using supercritical carbon dioxide extraction were also evaluated using RSM [29]. Liu et al. [30] successfully determined the optimum conditions for the extraction of antioxidant activity and yield from edible brown algae *Ascophyllum nodosum.* In another study, Yang et al. [31] optimized UAE to extract kinsenoside from plant *A. roxburghii* using RSM.

The present study utilized RSM for the first time to optimize the ultrasonic-assisted extraction (UAE) of the total phenolic content (TPC) and ABTS from edible lichen samples (*Evernia divaricata*, *Evernia prunastri*, *Bryoria fuscescens*, *Pseudevernia furfuracea*, and *Lobaria pulmonaria*). This study determined the effects of three variables (solvent concentration, extraction temperature, and extraction time) on establishing the optimal extraction conditions to enhance the TPC and ABTS activity of ultrasound-assisted lichen extracts. Additionally, using the optimal extraction conditions of UAE, lichen samples were assessed in terms of the changes in their individual phenolic, mineral, volatile, and antibacterial properties.

## 2. Materials and Methods

### 2.1. Chemicals and Reagents

Trolox (97%), 2,2-diphenyl-1-picrylhydrazyl (DPPH) radical, 2,2-azinobis (3-ethylbenzothiazoline-6-sulphonic acid) (ABTS), gallic acid standard, sodium carbonate, Folin–Ciocalteu’s (FC’s) phenol reagent, and other chemicals were obtained from Sigma-Aldrich and Merck LLC. (Steinheim, Germany).

### 2.2. Lichen Collection and Identification

Five species of lichens, *Evernia divaricata*, *Evernia prunastri*, *Pseudevernia furfuracea*, *Bryoria fuscescens*, and *Lobaria pulmonaria*, were collected from Türkiye. A total of 100 g of each lichen species was collected, and the location, altitude, and season of the collection are shown in Table 1. The determination of the lichen species was achieved by G. Ozyigitoglu using standard methods [32]. The morphological characteristics found via macroscopic and microscopic studies, as well as the basis of colorful responses using chemical reagents, were used to identify the collected lichens, and they were identified according to the references’ keys. The voucher specimens were preserved in the Herbarium of the Department of Biology at Marmara University and dried at room temperature.

### 2.3. Preparation of the Lichen Extracts

The dry lichen samples were ground using a spice grinder. The UAE process extracted powdered lichens using an ultrasonic bath (WiseClean, DH.WUC.D10H, Wertheim, Germany) continuously. Fourteen different experimental points were obtained using Design Expert Software (Version 7; Stat-Easy Co., Minneapolis, MN, USA), in which the extraction conditions, namely, extraction temperature (25–40 °C), time (5–20 min), and ethanol concentration (0–80%) are shown for each batch (Table S1). To assess the effect of solvent polarity on the extraction efficiency of bioactives, ethanol–water mixtures at concentrations of 0%, 40%, and 80% ethanol (*v*/*v*) were chosen. These concentrations were selected to represent a broad polarity spectrum. This approach aimed to capture a wide spectrum of phytochemicals with differing polarities, ensuring comprehensive extraction. Ethanol is a well-known food-grade solvent often used in the extraction of phytochemicals due to its efficacy and safety. Each batch of lichen powders (0.5 g) was put into glass tubes, followed by the addition of 10 mL of solvent. Sonication was conducted at varying extraction times, solvent concentrations (ethanol–water), and extraction temperatures for each sample. Following extraction, the lichens were centrifuged at 2250× *g* for 15 min. The extracts were ultimately filtered and concentrated utilizing the rotary evaporator (Heidolph, Schwabach, Germany) at 45 °C and stored at −20 °C.

### 2.4. Experimental Design

This study utilized RSM to assess three factors in two responses. A central composite design was employed to research the relationship between independent factors and a dependent variable. The independent variables (factors) were extraction temperature (°C, X_1_), extraction time (min, X_2_), and ethanol concentration (%, X_3_), and the responses (dependent variables) were the total phenolic content (TPC) and ABTS. After the preliminary tests, the experimental ranges of the independent variables were defined and are presented in Table S1. The effect of the process parameters on bioactives is determined using quadratic models. The model’s success was assessed using the coefficient of determination (R2), lack of fit, and the model’s *p*-value. All experimental points were analyzed in triplicate, with findings shown as mean values and standard deviations.

### 2.5. Assessment of Total Phenolic Content and Antioxidant Capacity

The total phenolic content (TPC) of the lichen extracts was calculated following the methods described by Singleton et al. [33]. Gallic acid was chosen as the Reference standard. In order to quantify the absorbance, a Shimadzu 150 UV-1800 spectrophotometer was used (Kyoto, Japan). The results are given as mg gallic acid equivalent (GAE)/g dry weight (dw).

The ABTS radical scavenging activity [34], the DPPH radical scavenging activity [35], and the copper-reducing antioxidant capacity (CUPRAC) [36] were assessed. The absorbance was determined at the following wavelengths: 734, 517, and 450 nm, respectively. Their findings were given as μmol Trolox equivalent (TE)/g dw.

### 2.6. Phenolic Profile of the Optimized Lichen Extracts

The phenolic profiles of optimized lichen extracts were evaluated using a HPLC system (SIL-20A HT autosampler, LC-20AD pump, DGU-20A5R degasser, CTO-10ASVP oven, and CMB-20 A communication module) coupled to a diode array detector, SPDM20A DAD (Shimadzu, Japan), according to Ozkan et al. [37]. Separations were performed at 40 °C on an Inertsil^®^ ODS C-18 reversed-phase column (250 × 4.6 mm × 5 μm, GL Sciences, Tokyo, Japan). The mobile phases used were acetic acid in water (Mobil A, 0.1:99.9, *v*/*v*) and acetic acid in acetonitrile (Mobil B, 0.1:99.9, *v*/*v*). Working solutions were prepared by diluting the stock solutions with the appropriate solvent to have 10–100 µg/mL. The calibration curves based on triplicate injections demonstrated good linearity, with R^2^ values exceeding 0.99 (peak area vs. concentration). The findings were given as mg/100 g dw.

### 2.7. Mineral Analysis

Mineral analysis of lichen samples was performed by following the method of Muthu et al. [38] and using the ICP-MS instrument (ICP-MS, 7700 Series x, Agilent, Tokyo, Japan). Before the analysis, the calibration standards, control standards, calibration blank solution, sample blank, and control samples were all prepared accordingly. The findings were given as mg/100 g dw.

### 2.8. GC–MS Analysis

GC–MS analyses of lichen extracts were conducted, with some modifications to the method of [39]. GC–MS analyses were performed using a Restec (Bellefonte, PA, USA) Rtx-5MS fused silica capillary column (30 m × 0.25 mm × 0.25 μm). The percentage content of the lichen extracts was calculated based on the GC peak regions. Volatile compounds were identified by comparing whole ion chromatograms to commercial mass spectra libraries (NIST27 and WILEY 7). The quantification of each volatile component was accomplished on the basis of the relative area of the total ion chromatogram (TIC) peaks of volatile components.

### 2.9. Antibacterial Activity

The bacterial strains used in this study were *Staphylococcus aureus* (ATCC 25923), *Salmonella* Typhimurium (ATCC 14028), and *Escherichia coli* O157: H7 (ATCC 33150). The pathogenic bacterial cultures were prepared in nutrient broth at 37 °C for 18 h. The minimum inhibitory concentration (MIC) values were evaluated in 96-well microplates utilizing the microdilution assay according to Ranković et al. [18] and Paudel et al. [40], with slight modifications. For antibacterial activity, optimized lichen extracts were used and lichen extracts were lyophilized. For analysis, 100 µL of bacterial culture was first added to the microplate wells, and then lichen extracts were added. The highest concentration, 100 mg/mL, was dissolved in ethanol and then dilutions of 5–100 mg/mL were prepared. The prepared stock solutions were sterilized using a 0.22 filter before analysis. Ethanol and nutrient broth were used as two different negative controls and all experiments were performed in triplicate. Antibiotic streptomycin was used for the positive controls. After incubation at 37 °C for 24 h, the IC_50_ values were defined as the concentration of the extract that inhibits 50% of the growth of the organism. The optical densities of the cultures were measured at a wavelength of 520 nm (iMark, Bio-Rad, Munich, Germany).

### 2.10. Statistical Analysis

All experiments were conducted in triplicate, and the findings were presented as the mean ± standard deviation. Statistical analysis was conducted using SPSS Statistics Software (IBM version 20.0., Armonk, NY, USA). A one-way analysis of variance (ANOVA) was conducted to compare the mean values of optimized lichen extracts, followed by Tukey’s post hoc test to assess the differences in bioactive qualities across the lichen samples.

## 3. Results and Discussion

### 3.1. Modeling and Optimization via RSM Model

The experiment was performed in fourteen runs to investigate the effects of the selected variables on the TPC and ABTS values obtained under different experimental conditions. The TPC values of all lichen extracts ranged from 0.80 to 16.90 mg/g dry weight, whereas the ABTS values varied from 1.37 to 319.76 mg/g dry weight. The maximum TPC and ABTS values were recorded for *E. divaricata* at an 80% ethanol concentration, 12.5 min, and temperatures of 25 and 40 °C. Cebi et al. [27] studied the experimental values of the TPC of cinnamon bark powder extract obtained via UAE in the experimental conditions (extraction temperature, ethanol concentration, and time) in 19 runs. The estimated coefficients of the polynomial equations’ quadratic, linear, and interaction components, as well as their significance (*p*-values), were assessed. For lichen samples, the linear and quadratic terms were very significant (*p* ˂ 0.01), according to Table 2. The lack of fit was utilized to evaluate the model’s suitability, and was not statistically significant (*p* > 0.05), indicating that the model effectively represented the experimental data. The quadratic ethanol concentration was found to be the most important process parameter in the UAE of both the TPC and ABTS from *E. divaricata* and *P. furfuracea* (*p* < 0.0001). As for the ABTS value in *B. fuscescens*, the quadratic effect of the solvent concentration was found to be the most significant parameter, followed by the extraction time and temperature. Bilgin et al. [41] also reported that the most effective process parameter was the solvent concentration of their natural plant extraction system. In a study by Ozcan et al. [42], the effect of the extraction time and temperature on the TPC, TFC, AA, and TAC values of the purple basil samples was expressed by the quadratic model. Also, the same author reported that the quadratic model successfully modeled the effect of temperature and time on the bioactive compound extraction yield with a high R^2^ value (0.99), and that the lack of fit was insignificant.

**Figure 1:**
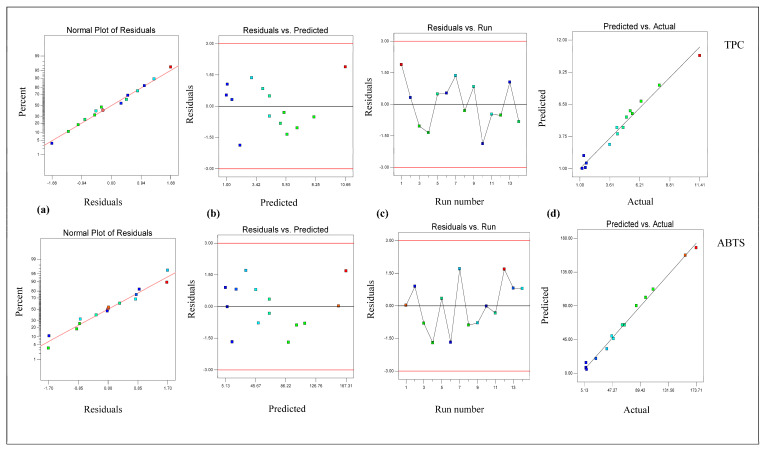
Plot of residuals for response TPC and ABTS in *B. fuscescens*. (**a**) Normal plot of the residuals, (**b**) residuals versus the predicted values, (**c**) residuals versus experimental run, and (**d**) predicted versus actual values.

**Figure 2:**
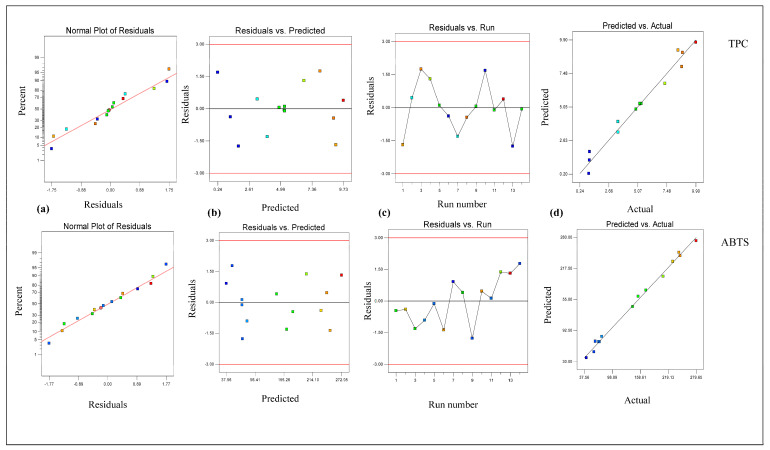
Plot of residuals for response TPC and ABTS in *E. divaricata*. (**a**) Normal plot of the residuals, (**b**) residuals versus the predicted values, (**c**) residuals versus experimental run, and (**d**) predicted versus actual values.

**Figure 3:**
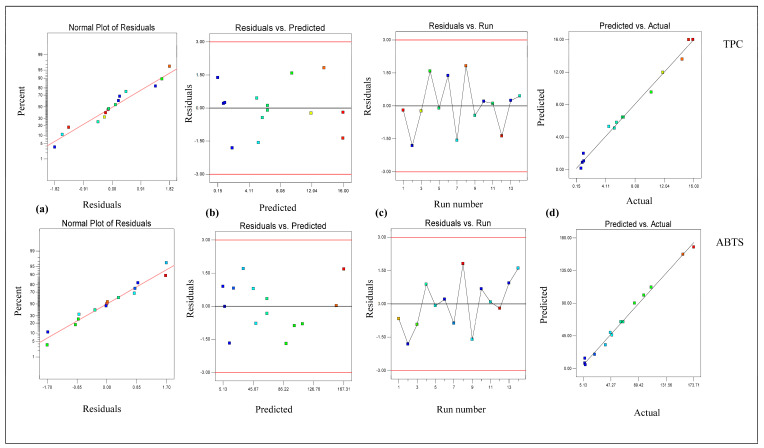
Plot of residuals for response TPC and ABTS in *E. prunastri*. (**a**) Normal plot of the residuals, (**b**) residuals versus the predicted values, (**c**) residuals versus experimental run, and (**d**) predicted versus actual values.

**Figure 4:**
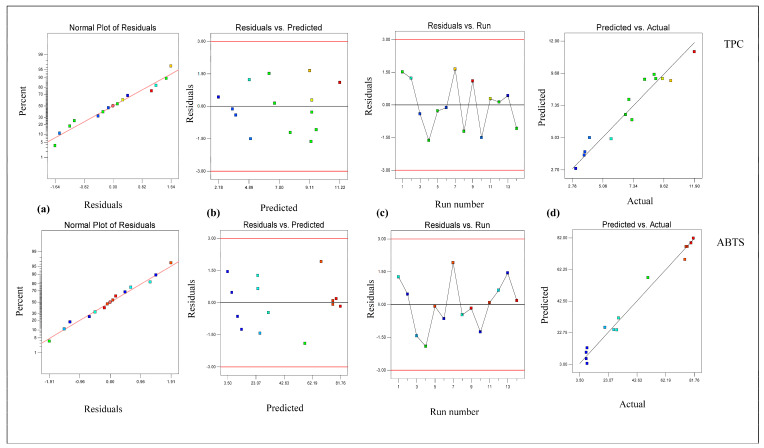
Plot of residuals for response TPC and ABTS in *L. pulmonaria*. (**a**) Normal plot of the residuals, (**b**) residuals versus the predicted values, (**c**) residuals versus experimental run, and (**d**) predicted versus actual values.

**Figure 5:**
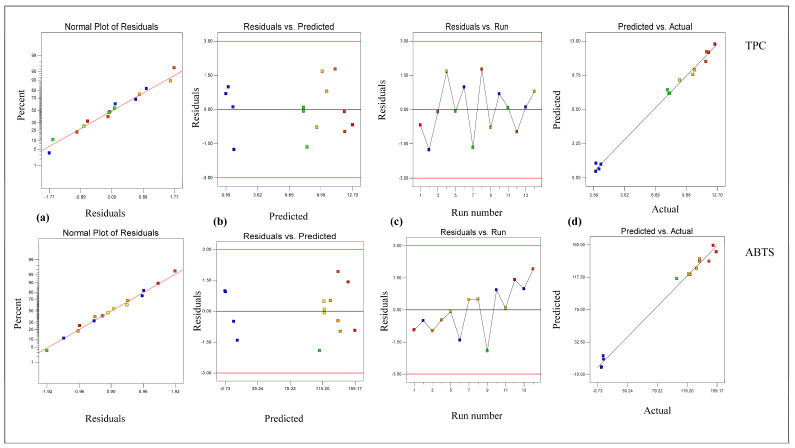
Plot of residuals for response TPC and ABTS in *P. furfuracea*. (**a**) Normal plot of the residuals, (**b**) residuals versus the predicted values, (**c**) residuals versus experimental run, and (**d**) predicted versus actual value.

From Table 2, it is clear that the linear terms (extraction temperature [X_1_] and ethanol conc. [X_3_]) and interaction term (extraction temperature × ethanol conc. [X_1_X_3_]) had considerable effects on the TPC value in *B. fuscescens*, the linear terms (extraction temperature [X_1_], extraction time [X_2_], and ethanol conc. [X_3_]) and quadratic term (ethanol concentration [X_3_ ^2^]) had considerable effects on the TPC value in *E. divaricata*, the linear terms (extraction temperature [X_1_] and ethanol conc. [X_3_]) had considerable effects on the TPC value in *E. prunastri*, the linear term (ethanol conc. [X_3_]) and quadratic term (ethanol concentration [X_3_ ^2^]) had considerable effects on the TPC value in *L. pulmonaria*, and the linear terms (extraction temperature [X_1_] and ethanol conc. [X_3_]) and quadratic term (ethanol concentration [X_3_ ^2^]) had considerable effects on the TPC value in *P. furfuracea*. According to Table 2, the R^2^ and adj R^2^ values ranged from 0.99 to 0.94 and from 0.98 to 0.89, respectively, for the TPC, and the lack of fit was insignificant (*p* > 0.05). This finding demonstrated that the quadratic model effectively described the effect of the extraction process parameters on the TPC and ABTS. In addition, the developed RSM for the TPC and ABTS was checked via residual analysis. The residual plots for the response parameters of the TPC and ABTS in lichen samples are given in Figure 1, Figure 2, Figure 3, Figure 4 and Figure 5. The data are virtually evenly distributed in a straight line in a normal plot of residuals (Figure 1a, Figure 2a, Figure 3a, Figure 4a and Figure 5a), indicating a strong correlation between the empirical and calculated values. Figure 1, Figure 2, Figure 3, Figure 4 and Figure 5 compare the residual values to the predictions, demonstrating that there was very little fluctuation between the fitted and observed values. Figure 1b, Figure 2b, Figure 3b, Figure 4b and Figure 5b compare the residual values to the predictions, demonstrating that there was very little fluctuation between the fitted and observed values. There were both negative and positive experimental runs, as seen with the residuals estimated versus the experimental runs in Figure 1c, Figure 2c, Figure 3c, Figure 4c and Figure 5c. Runs of positive and negative residuals were more likely to exist, which is explained by the existence of a particular association. As a result, Figure 1d, Figure 2d, Figure 3d, Figure 4d and Figure 5d show that the RSM model was sufficient.

The statistical optimization of the TPC and ABTS in UAE was conducted utilizing the RSM-generated model. The optimum extraction parameters were established according to the peak responses of the TPC and ABTS in lichen extracts. The extracts obtained via UAE are computed using the quadratic regression equation derived from the Design Expert 7.0 software. The optimum extraction conditions for five lichen samples are presented in Table 3. The optimum conditions were determined for *B. fuscescens* (40 °C, 19.34 min, and 80%), *E. divaricata* (32.23 °C, 20 min, and 80%), *E. prunastri* (37.02 °C, 20 min, and 80%), *L. pulmonaria* (25.20 °C, 20 min, and 49.49%), and *P. furfuracea* (38.04 °C, 19.63 min, 79.57%). The predicted values of the TPC in *B. fuscescens*, *E. divaricata*, *E. prunastri*, *L. pulmonaria*, and *P. furfuracea* were 10.92, 15.90, 9.89, 11.52, and 12.62 mg GAE/g dw, respectively. The predicted values of ABTS in *B. fuscescens*, *E. divaricata*, *E. prunastri*, *L. pulmonaria*, and *P. furfuracea* were 189.11, 311.85, 204.50, 81.11, and 163.75 µmol TE/g dw, respectively. It might be found that the experimental values highly matched the predicted values with a low error (Table 3). Similarly, the TPC values in *E. divaricata and L. pulmonaria* were found to be 11.8 and 8.24 mg GAE/g dw, respectively [43]. In a study by Stojković et al. [44], the TPC value of *E. prunastri* was found to be 22.11 mg GAE/g extract [44]. Cebi et al. [27] demonstrated that the most desirable solution for the maximization of the TPC corresponds to an extraction time of 49.98 min, ethanol concentration (*v*/*v*) of 60.71%, and temperature of 69.82 °C in cinnamon extract. Some studies have demonstrated that ultrasonic-assisted extraction could be a beneficial technique for obtaining bioactive substances. Also, a number of authors have talked about the mechanical disruption that ultrasound has on cell walls, which raises the concentration of a number of bioactive chemicals [45].

### 3.2. Antioxidant Capacities of the Optimized Lichen Extracts

Table S2 shows the values of DPPH and CUPRAC obtained from the UAE of lichen extracts prepared under optimal conditions. The DPPH values of the five lichen extracts changed from 7.19 to 26.04 μmol TE/g of dw, while the CUPRAC values of the eight lichen extracts ranged from 54.02 to 114.29 μmol TE/g of dw. *P. furfuracea* extract showed the highest DPPH and CUPRAC values. The CUPRAC values of both extracts in our investigation were higher than the results from the DPPH technique. Free radicals are formed in part by the Cu^2+^ ion; the reduction in the cupric ion suggests a different mechanism than in the DPPH technique, indicating antioxidant potential. This finding may be explained by the fact that, while the CUPRAC assay uses a reagent that is soluble in both aqueous and organic solvents and can measure both the hydrophilic and lipophilic antioxidant capacities of the extracts, the DPPH assay uses a radical that is only soluble in organic solvent, and may therefore more accurately represent the lipophilic antioxidants [46]. Similar results reported that *P. furfuracea* extract showed the best scavenging effect (DPPH) [4]. Similarly to our study, the extract of *P. furfuracea* showed a higher value than *E. prunastri* for the DPPH value [5]. Sarikurkcu et al. [47] reported that the DPPH and CUPRAC values in *P. furfuracea* methanol extract were 44.69 and 95.83 mg TE/g extract, respectively, and that these values were higher than those in our study. The differences in the results might be explained by differences in the extraction conditions and type of extraction solvent, post-harvesting conditions, environmental factors, and storage [48].

### 3.3. Phytochemical Compositions of the Optimized Lichen Extracts

The amounts of twenty-four individual compounds in the lichen extracts were determined via HPLC-DAD, and the results are shown in Table 4. Catechin was the major phenolic detected, followed by chlorogenic acid, ellagic acid, myricetin, chrysin, gallic acid, protocatechuic acid, quercetin, and kaempferol. Sarikurkcu et al. [47] reported the presence of hesperidin, p-hydroxybenzoic acid, and caffeic acid in the methanol and water extracts of *Pseudevernia furfuracea*. Myricetin was only determined in *B. fuscescens* (21.93 mg/100 g dw), *L. pulmonaria* (20.01 mg/100 g dw), and *P. furfuracea* (20.24 mg/100 g dw), whereas for all lichen extracts, other phenolics were also detected, but in low concentrations, such as *p*-coumaric acid, ferulic acid, and rutin (Table 4). *Evernia* species produce mainly evernic acid and usnic acid. Evernic acid was found to exhibit strong antimicrobial activity against different microorganisms, and anticancer activity against various cell lines [4]. Usnic acid plays many biological roles such as antibiotic, antifeedant, antimycotic, a photobiont regulator, and a UV filter [49]. Atranorin was detected only in *P. furfuracea.* Fumarprotocetraric acid was found exclusively in the sample of *B. fuscescens* and was not detected in any of the other samples. Atranorin and fumarprotocetraric acid showed interesting biological activities such as antioxidant, cytotoxicity, cytoprotective, antimicrobial, pro-apoptotic, and anticarcinogenic activities [50]. In addition, stictic acid was only detected in *L. pulmonaria.* In a study by Singh et al. [51], the phenolic contents of lichen samples changed between 0.03 and 970.01 and 0.16 and 1316.54 µg/g dw, respectively. The same authors reported that gallic acid was present in all the tested extracts, with the highest concentration found in the acetone and methanol extracts of *Flavoparmelia caperata,* and the maximum concentration of rutin was present in the acetone extract of *Lobaria retigera*. The same authors also reported that the most important phenolic in the methanol extract was kaempferol.

Ferulic acid, p-coumaric acid, chlorogenic acid, protocatechuic acid, gallic acid, phloridzin, rutin, vanillic acid, and syringic acid were detected in the water extract of *Usnea longissima* and, similarly to our study, chlorogenic acid (226.25 mg/kg) and gallic acid (123.79 mg/kg), as the major phenolic acids, were found at a higher level than other phenolics [52]. Gallic acid, ferulic acid, protocatechuic acid, *p*-coumaric acid, rutin, vanillic acid, and rutin were found in *Cladonia chlorophaea* (Florke ex Sommerf.) Sprengel, *Dermatocarpon miniatum* (L.) W. Mann, and *Parmelia saxatilis* (L.) Ach. [53]. In another study, *p*-coumaric acid, protocatechuic acid, ferulic acid, syringic acid, gallic acid, chlorogenic acid, rutin, and vanillic acid were found in the methanol and water extracts of *Peltigera canina* and *Umbilicaria nylanderiana* [54]. It has been noted that the presence of fumarprotocetraric acid has been considered an important diagnostic character, especially in *B. fuscescens s. lato* and *B. fuscescens* and *B. subcana* typically contain fumarprotocetraric acid in the soralia, medulla, and outer cortex of the thallus [55]. Additionally, Kosanić et al. [56] reported that the acetone extract of *E. prunastri* contained the metabolites atranorin, chloroatranorin, evernic acid, usnic acid, and physodic acid. Similarly, major metabolites identified in the extract *of P. furfuraceae* included 3-hydroxyphysodalic acid, physodalic acid, physodic acid, atranorin, and chloroatranorin [56].

### 3.4. Mineral Contents of Lichen Samples

Table 5 gives the mineral contents of the five lichen samples that have the highest phenolic compounds using the optimal extraction conditions of UAE. The levels of Mg and Ca in lichen species ranged from 1.07 to 124.70 mg/100 g dw and 22.62 to 701.94 mg/100 g dw, respectively, and were significantly different (*p* < 0.05) among the lichen extracts. The level of all minerals was higher in *E. prunastri* and *L. pulmonaria* samples compared to in other lichens. The level of Zn was not detected for *E. divaricata*, *B. fuscescens*, or *P. furfuracea.* Vinayaka et al. [57] reported that the content of Ca was the highest of all the elements in *Usnea pictoides*. A similar result was obtained by Kekuda et al. [58] for the lichen extracts of *Everniastrum cirrhatum*, where the content of Ca was the highest of all the elements. In our study, the level of K was abundant in *L. pulmonaria*. In a study by Muthu et al. [38], Ca was found to be the highest in all lichen extracts, followed by K. In a study by Storeheier et al. [59], lichens from the genera Cetraria and Cladonia contained Ca (0.2–2.1 g/kg dw), K (0.5–2.7 g/kg dw), Ca (0.2–2.1 g/kg dw), P (0.5–0.9 g/kg dw), Mg (0.1–0.5 g/kg dw), and Na (0.1–0.4 g/kg dw). Sodium (5.05 mg/100 g dw) was the highest in *L. pulmonaria* compared to in other lichens. The reason for the differences in microelements is probably due to variations in abiotic and biotic factors, the age of lichens, habitats, the solvent used for the extraction procedure, and the origin of plant materials [38]. Trace elements like Fe and Mn were also detected in all lichens, whereas Zn only was detected in *E. prunastri* and *L. pulmonaria.* Similarly to our study, in a study by Mokhtar et al. [60], the ranges of the concentrations of Zn and Mn in lichens were found to be 17.54–45.45 µg/g and 8.17–108.59 µg/g, respectively.

### 3.5. Determination of the Volatile Composition of Lichen Extracts

Lichens are known to produce a diverse range of biologically active compounds, both primary (intracellular) and secondary (extracellular) metabolites. Primary metabolites, essential for cell growth and maintenance, include polysaccharides, amino acids, proteins, vitamins, carotenoids, and polyols. In contrast, lichen secondary metabolites, although not contributing to growth, development, or reproduction, are typically derived from primary metabolism [61].

The GC–MS analysis of lichen extracts is presented in Table 6. A total of 19 compounds were identified *in E. divaricata* lichen extract. The most abundant components were 3-Benzenediol, 5-pentyl- (59.17%), 1-ethoxy-2-methoxy-4-methylbenzene (20.88%), and 5,6-Dimethoxy-1-indanone (9.36%). The proportions of other components were below 1%. In *B. fuscescens*, ribitol was the major volatile compound, followed by benzaldehyde, hexyl 2-methylbutanoate, and barbatolic acid. *B. fuscescens* was also found to be a good source of fatty acids composed of n-hexadecanoic acid and octadecanoic acid. The major volatile compounds identified in *P. furfuracea* were ribitol, mannitol, 2,5-dimethyl-4-hydroxy-3-hexanone, and 1,3-benzenediol, 5-pentyl-. Similarly to *B. fuscescens*, *P. furfuracea* extract contained different proportions of fatty acids, with 1% 9,12,15-octadecatrienoic acid and 3% 9,12-octadecadienoic acid, n-hexadecanoic acid, and octadecanoic acid. In *E. prunastri*, the most abundant compound identified was 3,5-dihydroxytoluene, followed by 1% ribitol, 2% 3-methoxy-2-methylphenol, and 3%. *E. prunastri* was found to contain certain proportions of fatty acids, although not to the same extent as A and B. Additionally, *E. prunastri* was found to contain a portion small of usnic acid. In *L. pulmonaria*, the most abundant volatile component is mannitol, followed by ribitol, Octadec-9-Enoic Acid, Ethyl Oleate, and Ethyl Linoleate. Polyols play a role in carbohydrate storage and serve to protect organisms from osmotic, salt, and oxidative stresses [62]. Additionally, polyols act as cryoprotectants, protecting lichens in cold habitats [63]. Among polyols, D-ribitol and D-mannitol are the most abundant in lichens [64]. D-ribitol is exported from algal photobionts to mycobionts [65], while D-mannitol is produced and metabolized by lichen fungi [66]. Olivetol is a phenolic compound primarily found in lichens. It has been identified as a substance produced by certain insects as an antiseptic and protective secretion [67]. Indeed, lichens contain a variety of fatty acids that are commonly found in higher plants [68]. The composition of fatty acids in lichens can vary depending on the species and cultivation conditions, such as temperature [69]. It is observed that major fatty acids in *Evernia mesomorpha* and *Parmelia* sp. include linoleic and oleic acids. Additionally, they contain linolenic acid as a minor component, a compound present in all photosynthesizing higher algae and green plants [70]. Temperature plays a pivotal role in influencing fatty acid metabolism. Research on lichens has demonstrated a seasonal variation in the degree of unsaturation, with a decrease as temperatures rise [71]. Notably, *Xanthoria parietina*, when thriving at higher elevations, exhibits a distinct behavior, showing an increase in the unsaturated fatty acid content in response to elevated temperatures and humidity levels [72]. The GC–MS analysis revealed the presence of numerous compounds in edible lichen extracts, with some components being highly abundant, while others are present in smaller proportions. Additionally, edible lichens serve as rich sources of polyols and fatty acids, with different species exhibiting varying compositions influenced by the type of species.

### 3.6. Antibacterial Activity of the Optimized Lichen Extracts

The antibacterial activity of lichen extracts was evaluated against three pathogenic bacteria strains: *S. typhimurium* (ATCC 14028), *S. aureus* (ATCC 25923), and *E. coli* O157: H7 (ATCC 33150), as shown in Table 7. Concentrations ranging from 5 to 100 mg/mL were tested, and the concentrations exhibiting 50% bacterial inhibition (IC_50_ values) were determined. All lichen extracts displayed antimicrobial properties against the tested bacteria, with IC_50_ values ranging from 0.63 to 1.88 mg/mL. *L. pulmonaria* extract generally exhibited the weakest inhibition effect against all bacteria, while *E. divaricata* was the most effective among the lichens.

This potent antimicrobial activity may be attributed to the abundance of antimicrobial substances, notably olivetol, evernic acid, and usnic acid. Olivetol has been reported to possess antibacterial properties against these S. aureus strains, and the antibacterial activities were attributed to their action on the cytoplasmic membrane [73]. Evernic acid demonstrated antibacterial activity against various microorganisms. For instance, it exhibited activity against *Klebsiella pneumoniae*, *Bacillus mycoides*, *B. subtilis*, and *Candida albicans* at MIC 0.25 mg/mL. Its MIC against *Escherichia coli* was reported to be 0.5 mg/mL, while against *Penicillium purpurescens*, *Aspergillus flavus*, *A. fumigatus*, and *P. verrucosum*, the MIC was 1 mg/mL [56]. It has been reported that evernic acid exhibits moderate similarity to folic acid synthesis inhibitors such as sulfamethoxazole and trimethoprim [74]. In a study by Aslan et al. [75], the antimicrobial effects of methanol extracts from various species, including lichens such as *E. divaricata* and *E. prunastri*, were investigated. *E. divaricata* extract demonstrated significant antibacterial activity, inhibiting 20 out of 30 tested bacteria, consistent with our findings. Conversely, *E. prunastri* was reported to exhibit antimicrobial effects against *E. coli*, but was ineffective against *Salmonella* and *S. aureus* strains. It was also reported that extracts obtained from *P. furfuraceae* exhibited moderate antibacterial and antifungal effects, with inhibition observed against the tested microorganisms at concentrations ranging from 1.56 to 12.5 mg/mL. On the other hand, extracts derived from *E. prunastri* displayed inhibitory activity against all tested microorganisms at higher concentrations [56]. Previous research has suggested that lichens may be more effective against Gram-positive bacteria compared to Gram-negative bacteria, possibly due to differences in bacterial morphology, cell wall structures, and permeability levels [18,76]. Our study also observed stronger effects of lichen extracts *on S. aureus*, a Gram-positive bacterium, compared to Gram-negative bacteria, except *for B. fuscescens*. Lichens are known to possess antimicrobial properties attributed to secondary metabolites such as phenolic compounds [77]. Consistently, our analysis revealed the highest total phenolic content in the *E. divaricata* lichen extract. This correlation between phenolic content and antimicrobial activity aligns with previous research [75]. Notably, the isolated components of the lichen demonstrated potent antimicrobial activity, with minimum inhibitory concentrations (MICs) ranging from 0.0008 to 1 mg/mL against the tested microorganisms. Among these components, physodic acid exhibited the strongest antimicrobial activity, effectively inhibiting all bacterial and fungal species even at extremely low concentrations. The results of this study also demonstrated that the commercial antibiotic streptomycin, used as a positive control, exhibited stronger antibacterial activity than the lichen extracts against *S. aureus* and *E. coli.* However, streptomycin exhibited limited or relatively weak activity against *S. typhimurium* and was less effective than all the tested lichen extracts, with the exception of *L. pulmonaria.* In parallel with our findings, it was confirmed in previous studies [78,79,80] that some lichens exhibited higher antibacterial properties than commercial antibiotics, depending on the extraction parameters (time, solvent, and temperature) and especially the species characteristics.

The choice of solvent used in extraction may influence microbial inhibition [77,81,82,83]. For instance, Kosanić and Ranković [81] investigated the antimicrobial properties of different lichen species using solvents such as acetone, methanol, and water. Methanol and acetone extracts exhibited the inhibition of microorganisms, whereas the aqueous extract did not. Variations in microbial inhibition among different lichen species are attributed to the presence of various components with antimicrobial activity, influenced by extract type, concentration, and pathogenic bacterial strain.

Considering all the present results, functionally, lichens play important roles in water and nutrient cycling and provide food, forage, and habitat to humans and many other organisms in terms of sustainability.

## 4. Conclusions

In this study, RSM was effectively used to estimate and optimize the TPC and ABTS values of ultrasound-assisted edible lichen extracts. The results showed that various factors (extraction temperature, extraction time, and ethanol concentration) had significant effects on the TPC values in different lichen species. The R^2^ and adjusted R^2^ values ranged from 0.99 to 0.94 and from 0.98 to 0.85, respectively, for the TPC, indicating the good fit of the quadratic model to the data. Furthermore, the lack of fit was insignificant (*p* > 0.05), confirming the model’s reliability. The experimental values were in close agreement with the predicted values, affirming the model’s effectiveness. The phenolic profiles of the optimized lichen extracts revealed catechin as the major phenolic compound, followed by chlorogenic acid, ellagic acid, myricetin, chrysin, gallic acid, protocatechuic acid, quercetin, and kaempferol. Evernic acid and usnic acid were found in *Evernia* species. Atranorin was detected only *P. furfuracea.* Fumarprotocetraric acid was found exclusively in sample *B. fuscescens* and was not detected in any of the other samples. Additionally, the mineral content and antibacterial activities of the five edible lichen species were compared. Calcium was found to be the highest in all lichens, followed by potassium. *L. pulmonaria* extract exhibited the lowest level of inhibition against all tested bacteria, whereas the most effective inhibitory extracts among the lichen species were *E. divaricata* and *E. prunastri*. The findings demonstrate that edible lichen extracts contain significant sources of polyphenolics and essential minerals. These bioactive compounds are known for their antioxidant, anti-inflammatory, and health-promoting properties. Due to their compositional character, edible lichens may be potential sources for functional food development and nutraceutical uses. Further research on edible lichen bioavailability, species selection, and processing methods is warranted for future investigations in food science and technology.

## Figures and Tables

**Table 1:** Lichen species, location (province), and collection date.

Species	Province	Altitude (m)	Collection Date
*Bryoria fuscescens*	Uludağ/Bursa	1772	7 October 2021
*Evernia divaricata*	Uludağ/Bursa	1772	7 October 2021
*Evernia prunastri*	Kartepe/Sakarya	750	28 August 2021
*Lobaria pulmonaria*	Kartepe/Sakarya	1111	11 June 2021
*Pseudevernia furfuracea*	Uludağ/Bursa	1216	6 October 2021

**Table 2:** RSM model statistics and ANOVA analysis.

	TPC					ABTS				
*B. fuscescens*										
Regression Coefficients	Sum of Squares	Df	Mean Square	F-Value	*p*-Value	Sum of Squares	Df	Mean Square	F-Value	*p*-Value
Model ^1^	101.61	9	11.29	17.69	0.0070, significant	36,274.55	9	4030.51	69.43	0.0005, significant
X_1_—Temperature	9.33	1	9.33	14.62	0.0187	1453.83	1	1453.83	25.04	0.0075
X_2_—Time	1.04	1	1.04	1.63	0.2702	1851.45	1	1851.45	31.89	0.0048
X_3_—Ethanol conc.	81.78	1	81.78	128.15	0.0003	30,154.76	1	30,154.76	519.46	<0.0001
X_1_X_2_	0.03	1	0.03	0.05	0.8264	184.63	1	184.63	3.18	0.1491
X_1_X_3_	7.87	1	7.87	12.34	0.0246	863.41	1	863.41	14.87	0.0182
X_2_X_3_	0.42	1	0.42	0.66	0.4620	624.93	1	624.93	10.77	0.0305
X_1_ ^2^	0.54	1	0.54	0.85	0.4081	324.50	1	324.50	5.59	0.0773
X_2_ ^2^	0.35	1	0.35	0.55	0.5004	0.50	1	0.50	0.01	0.9304
X_3_ ^2^	0.01	1	0.01	0.02	0.8900	595.43	1	595.43	10.26	0.0328
Residual	2.55	4	0.64			232.20	4	58.05		
Lack of Fit	2.40	3	0.80	5.41	0.3036, not significant	225.68	3	75.23	11.5415	0.2123, not significant
Pure Error	0.15	1	0.15			6.52	1	6.52		
Cor Total	104.16	13				36,506.75	13			
R^2^	0.9755					0.9936				
Adj—R^2^	0.9204					0.9793				
	TPC					ABTS				
*E. divaricata*										
Regression coefficients	Sum of squares	Df	Mean square	F-value	*p*-value	Sum of squares	Df	Mean square	F-value	*p*-value
Model ^1^	388.92	9.00	43.21	48.22	0.0010, significant	168,477.33	9	18,719.70	36.49	0.0017, significant
X_1_—Temperature	6.21	1.00	6.21	6.93	0.0580	3.50	1	3.50	0.01	0.9381
X_2_—Time	12.18	1.00	12.18	13.59	0.0211	3064.95	1	3064.95	5.97	0.0709
X_3_—Ethanol conc.	356.93	1.00	356.93	398.26	<0.0001	149,222.67	1	149,222.67	290.87	<0.0001
X_1_X_2_	3.87	1.00	3.87	4.32	0.1062	16.28	1	16.28	0.03	0.8673
X_1_X_3_	0.42	1.00	0.42	0.47	0.5309	936.07	1	936.07	1.82	0.2481
X_2_X_3_	2.44	1.00	2.44	2.73	0.1740	1841.28	1	1841.28	3.59	0.1311
X_1_ ^2^	0.52	1.00	0.52	0.59	0.4869	1.44	1	1.44	0.00	0.9603
X_2_ ^2^	0.57	1.00	0.57	0.64	0.4692	596.01	1	596.01	1.16	0.3418
X_3_ ^2^	5.10	1.00	5.10	5.69	0.0756	11,015.95	1	11,015.95	21.47	0.0098
Residual	3.58	4.00	0.90			2052.11	4	513.03		
Lack of Fit	3.57	3.00	1.19	116.40	0.0680, not significant	2048.62	3	682.87	195.96	0.0525, not significant
Pure Error	0.01	1.00	0.01			3.4848	1	3.48		
Cor Total	392.50	13.00				170,529.44	13			
R^2^	0.9908					0.9880				
Adj—R^2^	0.9703					0.9609				
	TPC					ABTS				
*E. prunastri*										
Regression coefficients	Sum of squares	Df	Mean square	F-value	*p*-value	Sum of squares	Df	Mean square	F-value	*p*-value
Model ^1^	118.13	9	13.13	17.07	0.0075, significant	90,207.35	9	10,023.04	96.31	0.0003, significant
X_1_—Temperature	4.98	1	4.98	6.48	0.0636	233.52	1	233.52	2.24	0.2085
X_2_—Time	2.82	1	2.82	3.67	0.1281	2023.39	1	2023.39	19.44	0.0116
X_3_—Ethanol conc.	107.82	1	107.82	141.04	0.0003	12,836.04	1	12,836.04	123.34	0.0004
X_1_X_2_	0.08	1	0.08	0.11	0.7588	1.66	1	1.66	0.02	0.9055
X_1_X_3_	1.98	1	1.98	2.57	0.1839	496.98	1	496.98	4.78	0.0942
X_2_X_3_	0.33	1	0.33	0.43	0.5475	70.95	1	70.95	0.68	0.4554
X_1_ ^2^	0.03	1	0.03	0.04	0.8432	952.91	1	952.91	9.16	0.0389
X_2_ ^2^	0.05	1	0.05	0.07	0.8109	155.26	1	155.26	1.49	0.2890
X_3_ ^2^	0.01	1	0.01	0.01	0.9158	66,753.37	1	66,753.37	641.42	<0.0001
Residual	3.08	4	0.77			416.29	4	104.07		
Lack of Fit	3.05	3	1.02	39.41	0.1164, not significant	414.61	3	138.20	82.53696151	0.0807, not significant
Pure Error	0.03	1	0.03			1.67	1	1.67		
Cor Total	121.20	13				90,623.63	13			
R^2^	0.9746					0.9954				
Adj—R^2^	0.9175					0.9851				
	TPC					ABTS				
*L. pulmonaria*										
Regression coefficients	Sum of squares	Df	Mean square	F-value	*p*-value	Sum of squares	Df	Mean square	F-value	*p*-value
Model ^1^	91.07	9	10.12	6.57	0.0427, significant	11.788.45	9	1309.83	25.15	0.0036, significant
X_1_—Temperature	1.20	1	1.20	0.78	0.4268	101.15	1	101.15	1.94	0.2359
X_2_—Time	1.49	1	1.49	0.97	0.3807	35.77	1	35.77	0.69	0.4539
X_3_—Ethanol conc.	8.56	1	8.56	5.56	0.0778	667.51	1	667.51	12.82	0.0232
X_1_X_2_	1.51	1	1.51	0.98	0.3779	307.13	1	307.13	5.90	0.0721
X_1_ X_3_	0.48	1	0.48	0.31	0.6071	0.11	1	0.11	0.00	0.9652
X_2_X_3_	3.37	1	3.37	2.19	0.2132	7.58	1	7.58	0.15	0.7222
X_1_ ^2^	3.94	1	3.94	2.56	0.1851	3.61	1	3.61	0.07	0.8054
X_2_ ^2^	1.13	1	1.13	0.74	0.4391	49.63	1	49.63	0.95	0.3843
X_3_ ^2^	61.32	1	61.32	39.82	0.0032	10,210.40	1	10,210.40	196.02	0.0002
Residual	6.16	4	1.54			208.35	4	52.09		
Lack of Fit	6.04	3	2.01	16.6072	0.1780, not significant	207.96	3	69.32	177.4777978	0.0551, not significant
Pure Error	0.12	1	0.12			0.39	1	0.39		
Cor Total	97.23	13				11,996.80	13			
R^2^	0.9367					0.9826				
Adj—R^2^	0.8941					0.9436				
	TPC					ABTS				
*P. furfuracea*										
Regression coefficients	Sum of squares	Df	Mean square	F-value	*p*-value	Sum of squares	Df	Mean square	F-value	*p*-value
Model ^1^	265.29	9	29.48	77.75	0.0004, significant	48,709.72	9	5412.19	48.38	0.0010, significant
X_1_—Temperature	2.94	1	2.94	7.76	0.0495	808.75	1	808.75	7.23	0.0547
X_2_—Time	0.12	1	0.12	0.32	0.6009	15.62	1	15.62	0.14	0.7277
X_3_—Ethanol conc.	237.56	1	237.56	626.62	<0.0001	38,764.59	1	38,764.59	346.50	<0.0001
X_1_X_2_	0.46	1	0.46	1.22	0.3318	7.19	1	7.19	0.06	0.8124
X_1_X_3_	0.19	1	0.19	0.51	0.5135	99.49	1	99.49	0.89	0.3991
X_2_X_3_	0.04	1	0.04	0.11	0.7547	152.68	1	152.68	1.36	0.3076
X_1_ ^2^	1.44	1	1.44	3.79	0.1234	18.23	1	18.23	0.16	0.7071
X_2_ ^2^	1.75	1	1.75	4.63	0.0979	59.68	1	59.68	0.53	0.5056
X_3_ ^2^	16.31	1	16.31	43.01	0.0028	7849.48	1	7849.48	70.16	0.0011
Residual	1.52	4	0.38			447.50	4	111.87		
Lack of Fit	1.51	3	0.50	210.9378	0.0506, not significant	446.68	3	148.89	182.5703	0.0543, not significant
Pure Error	0.00	1	0.00			0.82	1	0.82		
Cor Total	266.80	13				49,157.21	13			
R^2^	0.9943					0.9909				
Adj—R^2^	0.9815					0.9704				

Df—degree of freedom values of “Prob > F” less than 0.05 indicate model terms are significant. ^1^; The model is a quadratic model. ^2^; quadratic term of temperature (X_1_ ^2^), time (X_2_ ^2^), and ethanol conc. (X_3_ ^2^). TPC: total phenolic content; ABTS: ABTS 2,2′-azino-bis (3-ethyl benzothiazoline6-sulphonic acid).

**Table 3:** Predicted and experimental values of the dependent variables at optimum conditions.

	Optimal Level of Process Parameters			Optimized Values (Predicted Values)		Experimental Values	
	Temperature (°C)	Time (min)	Ethanol Conc. (%)	TPC mg GAE/g dw	ABTS µmol TE/g dw	TPC mg GAE/g dw	ABTS µmol TE/g dw
*B. fuscescens*	40	19.34	80	10.92	189.11	11.89 ± 0.05	188.92 ± 0.80
*E. divaricata*	32.23	20	80	15.90	311.85	15.17 ± 0.13	311.38 ± 5.29
*E. prunastri*	37.02	20	80	9.89	204.50	10.97 ± 0.12	209.24 ± 0.97
*L. pulmonaria*	25	20	49.49	11.52	81.11	11.73 ± 0.13	80.33 ± 0.58
*P. furfuracea*	38.04	19.63	79.57	12.62	163.75	12.08 ± 0.15	165.98 ± 1.79

TPC: total phenolic content; ABTS: ABTS 2,2′-azino-bis (3-ethyl benzothiazoline6-sulphonic acid); GAE: gallic acid equivalent; TE: Trolox equivalent; and dw: dry weight.

**Table 4:** The concentration (mg/100 g dw) of phenolic compounds obtained from the optimum conditions of UAE of lichens.

	*B. fuscescens*	*E. divaricata*	*E. prunastri*	*L. pulmonaria*	*P. furfuracea*
Gallic acid	18.02 ± 0.18 ^a^	16.17 ± 0.07 ^c^	16.29 ± 0.02 ^bc^	15.99 ± 0.16 ^d^	16.47 ± 0.16 ^b^
Protocatechuic acid	12.16 ± 0.02 ^b^	nd	11.81 ± 0.17 ^c^	16.47 ± 0.41 ^a^	11.71 ± 0.04 ^c^
Catechin	42.52 ± 0.24 ^a^	10.08 ± 1.96 ^b^	nd	3.42 ± 0.08 ^c^	nd
P-hydroxybenzoic acid	nd	3.38 ± 0.04 ^b^	6.74 ± 0.24 ^a^	3.03 ± 0.08 ^c^	0.59 ± 0.02 ^d^
Syringic acid	1.22 ± 0.02 ^b^	0.57 ± 0.05^c^	1.66 ± 0.09 ^b^	5.76 ± 0.36 ^a^	nd
Ellagic acid	nd	nd	4.18 ± 0.17 ^d^	31.77 ± 0.48 ^a^	10.56 ± 0.43 ^b^
M-coumaric acid	1.70 ± 0.06 ^c^	1.05 ± 0.10 ^d^	5.47 ± 0.47 ^a^	4.47 ± 0.05 ^b^	nd
O-coumaric acid	0.33 ± 0.03 ^d^	4.01 ± 0.88 ^a^	2.43 ± 0.21 ^b^	0.40 ± 0.07 ^c^	nd
Chrysin	19.42 ± 0.45 ^a^	0.70 ± 0.04 ^d^	nd	10.33 ± 0.03 ^b^	2.63 ± 0.17 ^c^
Caffeic acid	3.04 ± 0.07 ^b^	2.53 ± 0.06 ^c^	2.97 ± 0.06 ^bc^	5.47 ± 0.03 ^a^	2.53 ± 0.03 ^c^
P-coumaric acid	0.31 ± 0.01 ^b^	0.38 ± 0.01 ^ab^	nd	0.21 ± 0.04 ^c^	0.40 ± 0.01 ^a^
Ferulic acid	1.27 ± 0.09 ^b^	0.93 ± 0.01 ^d^	1.03 ± 0.06 ^bc^	2.34 ± 0.04 ^a^	0.98 ± 0.09 ^c^
Myricetin	21.93 ± 1.37 ^a^	nd	nd	20.01 ± 0.06 ^b^	20.24 ± 0.15 ^b^
Quercetin	10.58 ± 0.01 ^a^	10.06 ± 0.17 ^a^	9.79 ± 0.01 ^bc^	9.57 ± 0.04 ^c^	9.94 ± 0.46 ^b^
Kaempferol	3.83 ± 0.01 ^b^	4.82 ± 0.01 ^a^	3.71 ± 0.03 ^c^	3.81 ± 0.05 ^b^	3.70 ± 0.02 ^c^
Chlorogenic acid	39.59 ± 0.15 ^a^	1.38 ± 0.21 ^b^	0.59 ± 0.06 ^c^	1.37 ± 0.16 ^b^	0.51 ± 0.05 ^c^
Rutin	0.68 ± 0.01 ^c^	1.15 ± 0.04 ^b^	nd	0.32 ± 0.06 ^d^	1.31 ± 0.03 ^a^
Sinapic acid	0.55 ± 0.04 ^b^	nd	nd	1.87 ± 0.12 ^a^	0.27 ± 0.01 ^c^
Evernic acid	nd	1350.77 ± 71.30 ^b^	1587.08 ± 11.33 ^a^	nd	nd
Usnic acid	nd	16.05 ± 0.85 ^b^	18.86 ± 0.13 ^a^	nd	nd
Stictic acid	nd	nd	nd	908.92 ± 28.36 ^b^	nd
Fumarprotocetraric acid	790.60 ± 17.78 ^a^	nd	nd	nd	nd
Physodic acid	nd	nd	nd	nd	351.41 ± 1.02 ^a^
atranorin	nd	nd	nd	nd	974.40 ± 1.17 ^a^

nd: not detected. Values are means ± standard deviation. ^a–d^ Means within the same row with different letters are significantly different at *p* < 0.05.

**Table 5:** The mineral analysis (mg/100 g dw) of lichen species.

	*B. fuscescens*	*E. divaricata*	*E. prunastri*	*L. pulmonaria*	*P. furfuracea*
Mg	1.07 ± 0.12 ^b^	1.21 ± 0.10 ^b^	124.70 ± 4.07 ^a^	118.77 ± 3.46 ^a^	1.28 ± 0.10 ^b^
Ca	22.62 ± 0.37 ^c^	30.54 ± 0.49 ^c^	701.94 ± 6.29 ^a^	546.58 ± 8.91 ^b^	41.58 ± 0.29 ^c^
Na	0.19 ± 0.03 ^c^	nd	4.17 ± 0.35 ^b^	5.05 ± 0.29 ^a^	nd
K	6.50 ± 0.22 ^c^	6.04 ± 0.15 ^c^	247.36 ± 6.19 ^b^	539.45 ± 16.60 ^a^	9.11 ± 0.14 ^c^
P	1.09 ± 0.07 ^c^	1.58 ± 0.12 ^c^	86.42 ± 0.47 ^b^	126.63 ± 4.97 ^a^	2.05 ± 0.18 ^c^
Fe	0.60 ± 0.01 ^c^	1.15 ± 0.13 ^c^	152.14 ± 1.95^a^	84.06 ± 0.64 ^b^	1.43 ± 0.14 ^c^
Zn	nd	nd	2.04 ± 0.11 ^b^	2.88 ± 0.27 ^a^	nd
Mn	0.18 ± 0.01 ^c^	0.21 ± 0.03 ^c^	12.06 ± 0.38 ^b^	20.95 ± 0.29 ^a^	0.12 ± 0.01 ^c^

nd: not detected. Values are means ± standard deviation. ^a–c^ Means within the same row with different letters are significantly different at *p* < 0.05.

**Table 6:** Compound characterization through GC–MS analysis of lichen extracts.

*Evernia divaricata*		
Retention Time	Name of Compound	% of Area
5.369	Cis-Ocimene	0.72
7.327	Bornylene	0.92
7.403	1,8-Cineole	0.33
9.566	Cyclopentasiloxane, Decamethyl-	1.02
12.274	Dodecamethylcyclohexasiloxane	0.52
14.591	Tetradecamethylcycloheptasıloxane	0.22
14.727	1-Ethoxy-2-Methoxy-4-Methylbenzene	20.88
15.482	1,3-Benzenediol, 5-Pentyl- (Olivetol)	59.17
15.990	4H-Pyran-4-One, 2,6-Dimethyl-	0.11
16.729	Cis-9-Tetradecen-1-Ol	0.58
18.268	5,6-Dimethoxy-1-İndanone	9.36
18.375	Octadecamethylcyclononasıloxane	0.67
19.779	N-Hexadecanoic Acid	1.58
19.927	Eıcosamethylcyclodecasıloxane	0.83
21.354	Tetracosamethylcyclododecasıloxane	0.49
22.643	Octadecamethylcyclononasıloxane	0.43
23.829	1H-Purin-6-Amine, [(2-Fluorophenyl)Methyl]-(CAS)	0.58
25.035	Tetracosamethylcyclododecasıloxane	0.45
28.431	Squalene	1.08
42.419	Glycerıne-1-Oleate-3-Palmıtate	0.06
*Bryoria fuscescens*		
Retention time	Name of Compound	% of Area
3.075	Propene 3,3,3-D3	0.4
3.177	Formamide, N-(2-Methylpropyl)-(CAS) N-Isobutylformamide	0.28
7.135	L-Limonene	0.31
8.525	Hexanal (CAS) N-Hexanal	0.3
9.501	Cyclopentasiloxane, Decamethyl-(CAS) Dimethylsiloxane Pentamer	0.35
14.366	1-Dodecanol (CAS) N-Dodecanol	0.46
14.75	1-Ethoxy-2-Methoxy-4-Methylbenzene	0.45
15.210	4-Cyano-2,2,5,5-Tetramethyl-3-İmidazoline-3-Oxide-1-Oxile	0.34
15.320	Dipropyl Hydroxybutanedioate	0.42
15.506	Benzaldehyde, 2,4-Dihydroxy-6-Methyl-(CAS) 2,4-Dihydroxy-6-Methylbenzaldehyde	16.61
15.670	1-Heptanethiol (Cas) Heptylthiol	2.53
15.890	Hexyl 2-Methylbutanoate	13.89
16.113	Ribitol	35.61
16.730	Cis-9-Tetradecen-1-Ol	1.09
17,331	Benzoic Acid, 2,4-Dihydroxy-3,6-Dimethyl-, Methyl Ester (CAS) Methyl 2,4-Dihydroxy-3,6-Dimethylbenzoate	0.29
17,601	Cyclohexene, 1-(3-Ethoxy-1-Propenyl)-, (Z)-(CAS) CIS-1-(1-CYCLOHEXENYL)-3-ETHOXYPROPENE	0.69
17.71	Tetradecanoic Acid (CAS) Myristic Acid	0.55
17.78	Ethyl 2,4-Dihydroxy-6-Methylbenzoate	0.69
18.398	Isopropyl Myristate	0.33
18.749	Barbatolic Acid	6.35
19.524	1,4,8-Dodecatriene, (E,E,E)-(Cas)	0.32
19.6	Ethanol, 2-(9-Octadecenyloxy)-, (Z)-(CAS) 2-CIS-9-OCTADECENYLOXY ETHANOL	0.55
19.781	N-Hexadecanoic Acid	2.5
20.01	Phthalıd, 4,6-Dımethoxy-	0.46
20.11	Hexadecanoic Acid, Ethyl Ester (CAS) Ethyl Palmitate	0.27
20.547	5-Hydroxymethyl-1,1,4a-Trımethyl-6-Methylene-Decahydro-Naphthalen-2-Ol	0.33
21.271	(E)-Phytol	0.34
21.451	9,12-Octadecadienoic Acid (Z,Z)-	1.86
21.489	Octadec-9-Enoıc Acıd	3.97
21.679	Octadecanoic Acid	1.34
22.86	Cyclohept-4-Enecarboxylıc Acıd	0.43
24.396	Benzoesaeure, 5-Methyl-2-Trımethylsılyloxy-, Trımethylsılylester	0.27
24.701	Hexadecanoic Acid, 2-Hydroxy-1-(Hydroxymethyl)Ethyl Ester (CAS) 2-Monopalmitin	0.33
25.075	1,2-Benzenedicarboxylic Acid, Diisooctyl Ester	0.4
26.889	Octadecanoic Acid, 2,3-Dihydroxypropyl Ester (CAS) 1-Monostearin	2.65
27.287	1,2-Benzenedicarboxylic Acid, Dioctyl Ester (CAS) Dioctyl Phthalate	0.42
27.49	1,3(2H,9bh)-Dibenzofurandione, 2,6-Diacetyl-7,9-Dihydroxy-8,9b-Dimethyl-(CAS) Usno	0.54
28.43	Squalene	0.34
38.542	D-Mannıtol, 1-Decylsulfonyl-	0.4
42.28	Cyclotrisiloxane, Hexamethyl-	0.34
*Pseudevernia furfuracea*		
Retention Time	Name of Compound	% of Area
9.527	Cyclopentasiloxane, Decamethyl-(CAS) Dimethylsiloxane Pentamer	0.23
12.268	Cyclohexasiloxane, Dodecamethyl-(CAS) Dodecamethylcyclohexasiloxane	0.15
14.015	Xanthosine (CAS) Xanthine Riboside	0.35
15.435	2-Ethyl-Norborneol	0.37
15.56	1,2-Ethanediol, 1-(2-Phenyl-1,3,2-Dioxaborolan-4-Yl)-, [S-(R*,R*)]-(CAS) L-THREIT, 1,2-O-(PHENYLBORANDIYL)-	0.64
15.605	Ribitol	0.65
15.735	1-Methylcyclohexanol	2.35
15.825	D-Mannitol	2.62
15.89	L-Arabinitol	2.41
15.93	Pentane-1,2,3,4,5-Pentaol	1.19
15.965	Myo Inosıtols	1.69
16.105	D-Mannitol	7.21
16.244	D-Mannitol	9.48
16.345	2,5-Dimethyl-4-Hydroxy-3-Hexanone	8.19
16.387	Ribitol	3.6
16.495	Ribitol	20.2
16.727	1,13-Tetradecadiene	0.9
17.252	1,3-Benzenediol, 4-Hexyl-(CAS) Oxana	1.15
17.321	Benzoic Acid, 2,4-Dihydroxy-3,6-Dimethyl-, Methyl Ester (CAS) Methyl 2,4-Dihydroxy-3,6-Dimethylbenzoate	3.05
17.805	1,3-Benzenediol, 5-Pentyl-	4.24
17.98	5,6-Decanediol (CAS)	0.26
18.377	Octadecamethylcyclononasıloxane	0.16
18.563	Neophytadıene	0.12
19.521	1,4,8-Dodecatriene, (E,E,E)-(CAS)	1
19.594	3,6-Octadecadienoic Acid, Methyl Ester (CAS) METHYL 3,6-OCTADECADIENOATE	0.58
19.779	N-Hexadecanoic Acid	3.86
21.27	Phytol Isomer	0.51
21.449	9,12-Octadecadienoic Acid (Z,Z)-	4.12
21.516	9,12,15-Octadecatrienoic Acid, Methyl Ester, (Z,Z,Z)-	6.9
21.673	Octadecanoic Acid	1.16
22.862	Atis-16-Ene, (5.Beta.,8.Alpha.,9.Beta.,10.Alpha.,12.Alpha.)- (CAS) Atiserene	0.14
23.148	2,6-Dı-Tert-Butyl-Octahydro-Azulene-3a,8-Dıol	2.3
23.384	7-Isopropyl-10-Methyl-1,5-Dıoxaspıro [5.5]Undecan-2,4-Dione	0.14
23.505	Benzyl Alectoronate	2.82
23.68	6,8-Dıoxabıcyclo(3.2.1)Octan-3.Beta.-Ol	0.19
24.317	1-Phenanthrenecarboxylic Acid, 1,2,3,4,4a,9,10,10a-Octahydro-1,4a-Dimethyl-7-(1-Methylethyl)-, [1R-(1.Alpha.,4a.Beta.,10a.Alpha	0.17
24.69	Hexadecanoic Acid, 2-Hydroxy-1-(Hydroxymethyl)Ethyl Ester (CAS) 2-Monopalmitin	0.81
25.56	4,7-Methanoisobenzofuran-1-Ol, 1,3,3a,4,7,7a-Hexahydro- (CAS) 2,2-DIMETHYL-1-(3-OXO-BUT-1-ENYL)-CYCLOPENTANECARBALDEHYDE	0.45
26.87	Octadecanoic Acid, 2,3-Dihydroxypropyl Ester	3.64
*Evernia prunastri*		
Retention Time	Name of Compound	% of Area
7.188	Dl-Limonene	0.1
7.263	1,8-Cineole	0.12
9.512	Cyclopentasiloxane, Decamethyl-(CAS) Dimethylsiloxane Pentamer	0.26
12.257	Cyclohexasiloxane, Dodecamethyl-	0.34
12.312	3-Methoxy-2-Methylphenol	5.82
13.067	3,5-Dihydroxytoluene	32.53
13.435	2-Methoxy-5-Methyl Pyrazıne	0.4
13.495	3,5-Dihydroxytoluene	0.22
14.583	Tetradecamethylcycloheptasıloxane	0.22
15.7	S-Methyl-L-Cysteine	0.18
15.76	Ribitol (CAS) Adonit	0.37
15.875	Sorbitol	1.41
15.92	Ribitol	0.71
16	D-Arabitol	2.06
16.085	Sorbitol	3.17
16.145	D-Arabitol	2.46
16.319	Ribitol (CAS) Adonit	8.83
16.355	Pentane-1,2,3,4,5-Pentaol	2.96
16.432	Ribitol (CAS) Adonit	9.22
16.717	Oleyl Alcohol	0.35
16.804	Methyl Ester Of 2-Hydroxy-4-Methoxy-6-Methyl-Benzoic Acid	6.2
17.296	Benzoic Acid, 2,4-Dihydroxy-3,6-Dimethyl-, Methyl Ester (CAS) Methyl 2,4-Dihydroxy-3,6-Dimethylbenzoate	1.62
17.59	Benzoic Acid, 2-Hydroxy-4-Methoxy-3,6-Dimethyl-, 4-Carboxy-3-Hydroxy-5-Methylphenyl Ester (CAS). Beta.-Resorcylic Acid, 6-Methy	0.28
18.367	Octadecamethylcyclononasıloxane	0.14
19.511	1,4,8-Dodecatriene, (E,E,E)-(CAS)	0.5
19.59	Tetradecadien-4,9 Ol-1	0.16
19.765	Pentadecanoic Acid	2.61
19.919	Eıcosamethylcyclodecasıloxane	0.23
20.963	1-Eicosanol (CAS) N-Eicosanol	0.39
21.259	Phytol	0.28
21.438	9,12-Octadecadienoic Acid (Z,Z)-	3.89
21.478	Octadec-9-Enoıc Acıd	3.3
21.506	11,14,17-Eicosatrienoic Acid, Methyl Ester (CAS) METHYL-11,14,17-EICOSATRIENOATE	3.72
21.663	Octadecanoic Acid	1.05
22.081	Acetic Acid, Octadecyl Ester	0.59
22.782	1-Octadecanol (CAS) Stenol	0.09
24.681	Hexadecanoic Acid, 2-Hydroxy-1-(Hydroxymethyl)Ethyl Ester (CAS) 2-Monopalmitin	0.58
25.061	1,2-Benzenedicarboxylic Acid, Bis(2-Ethylhexyl) Ester (CAS) Bis(2-Ethylhexyl) Phthalate	0.19
26.859	Octadecanoic Acid, 2-Hydroxy-1-(Hydroxymethyl)Ethyl Ester (CAS) 2-Monostearin	1.71
27.428	Usnic Acid	0.74
*Lobaria pulmonaria*		
Retention Time	Name of Compound	% of Area
9.224	5-Heptenoic Acid, Ethyl Ester, (E)-(CAS) ETHYL TRANS 5-HEPTENOATE	0.21
12.256	Cyclohexasiloxane, Dodecamethyl-	0.26
12.375	3-Methoxy-2-Methylphenol	0.32
12.432	Methyl 2-Oxo-5-Cycloheptene Carboxylate	0.49
13.975	P-Mentha-6,8-Dien-2-One, Semicarbazone (CAS) CARVONE SEMICARBAZONE	0.19
14.582	Tetradecamethylcycloheptasıloxane	0.22
15.215	3,3-Dimethyl-1,4-Diphenylazetidin-2-İmine	0.21
15.27	D-Mannitol	0.28
15.345	Sorbitol	0.76
16.255	D-Mannitol	51.09
16.315	Ribitol	16.59
16.617	Hexadecamethylcyclooctasıloxane	1.2
16.715	1-Piperazinecarboxylic Acid, Ethyl Ester	0.7
16.82	Spiro[5.5]Undec-8-En-1-One (CAS) Spiro[5.5]Undec-2-En-7-One	0.48
16.925	Sorbitol	0.44
18.032	(-)-Loliolide	0.17
18.365	Octadecamethylcyclononasıloxane	0.2
19.595	9,12,15-Octadecatrienoic Acid, Methyl Ester, (Z,Z,Z)-	0.44
19.765	N-Hexadecanoic Acid	2.53
19.919	Eıcosamethylcyclodecasıloxane	0.73
20.089	Hexadecanoic Acid, Ethyl Ester (CAS) Ethyl Palmitate	0.19
21.344	Tetracosamethylcyclododecasıloxane	0.18
21.436	9,12-Octadecadienoic Acid (Z,Z)-	2.72
21.475	Octadec-9-Enoıc Acıd	6.93
21.694	Ethyl Linoleate	3.34
21.737	Ethyl Oleate	4.71
22.631	Octadecamethylcyclononasıloxane	0.27
23.817	Eıcosamethylcyclodecasıloxane	0.25
24.031	2-Propen-1-One, 1,3-Diphenyl-	0.2
24.686	Hexadecanoic Acid, 2-Hydroxy-1-(Hydroxymethyl)Ethyl Ester (CAS) 2-Monopalmitin	0.32
25.026	1H-Purin-6-Amine, [(2-Fluorophenyl)Methyl]-(CAS)	0.42
26.504	Tetracosamethylcyclododecasıloxane	0.28
26.866	Octadecanoic Acid, 2,3-Dihydroxypropyl Ester	0.42
28.402	1H-Purin-6-Amine, [(2-Fluorophenyl)Methyl]-(CAS)	0.25
30.911	Eıcosamethylcyclodecasıloxane	0.47
34.295	Eıcosamethylcyclodecasıloxane	0.51
38.86	Cyclobuta[1,2:3,4]Dicyclooctene, Hexadecahydro-, (6a.Alpha.,6b.Alpha.,12a.Alpha.,12b.Alpha.)-(CAS) TRICYCLO[8.6.0.0(2,9)]HEXAD	0.34
38.926	1H-Purin-6-Amine, [(2-Fluorophenyl)Methyl]-(CAS)	0.36
40.61	Acetamide, N,N′-[(3.Beta.)-18-Hydroxypregn-5-Ene-3,20-Diyl]Bis- (CAS) 3.BETA.,20-BIS(ACETYLAMINO)-5-PREGNEN-18-OL	0.16
43.773	9-Nonylphenyl-3,6,9-Trioxanonanol, Mix of İsomers	0.17

**Table 7:** Antibacterial activity of lichen species in IC_50_ (mg/mL).

Lichen Species	IC_50_ (mg/mL)		
	*S. aureus* (ATCC 25923)	*E. coli* O157: H7 (ATCC 33150)	*S. typhimurium* (ATCC 14028)
*E. divaricata*	0.63 ± 0.02 ^e^	0.88 ± 0.03 ^c^	0.88 ± 0.09 ^d^
*E. prunastri*	0.80 ± 0.10 ^c^	0.99 ± 0.09 ^b^	0.90 ± 0.04 ^d^
*L. pulmonaria*	1.68 ± 0.08 ^a^	-	1.88 ± 0.04 ^a^
*P. furfuracea*	0.75 ± 0.01 ^d^	0.88 ± 0.06 ^c^	0.89 ± 0.07 ^e^
*B. fuscescens*	1.01 ± 0.08 ^b^	1.27 ± 0.09 ^a^	0.99 ± 0.03 ^c^
Streptomycin	0.49 ± 0.01 ^f^	0.51 ± 0.07 ^d^	1.76 ± 0.04 ^b^

The results are given as mean ± standard deviation of triplicate measurements. IC_50_: a 50% inhibition of bacterial growth. ^a–f^ Means within the same column with different letters are significantly different at *p* < 0.05.

## Data Availability

The original contributions presented in the study are included in the article/Supplementary Material, further inquiries can be directed to the corresponding author/s.

## Supplementary Materials

The following supporting information can be downloaded at: https://www.mdpi.com/article/10.3390/foods14152562/s1, Table S1: Independent variables and levels of variables for the Box Behnken design; Table S2: Antioxidant capacities obtained from optimum conditions of UAE of lichen samples.
