# Supplementary material for: Optimizing the Extraction of Polyphenols from Different Edible Lichens Using Response Surface Methodology and the Determination of Their Mineral and Antibacterial Properties
Source: Foods. 2025 Jul 22;14(15):2562. doi: 10.3390/foods14152562 (PMC12346634; doi:10.3390/foods14152562)
Supplement: Supplementary file 1 [file foods-14-02562-s001.zip › foods-3741300-supplementary.pdf]

Table S1. Independent variables and levels of variables for the Box Behnken design

Independent variables	Symbols	Coded level		
		Low level (-1)	Center value (0)	High level (+1)
Temperature (ºC)	X_1	25	32.5	40
Time (min)	X_2	5	12.5	20
Ethanol conc. (v/v%)	X_3	0	40	80

Table S2. Antioxidant capacities obtained from optimum conditions of UAE of lichen samples

	**DPPH (µmol TE/g dw)**	**CUPRAC (µmol TE/g dw)**
B. fuscescens	10.28±0.33	111.51±1.20
E. divaricata	7.36±0.51	54.20±1.76
E. prunastri	7.19±0.20	68.17±0.55
L. pulmonaria	11.03±0.17	54.02±1.06
P. furfuracea	26.04±0.92	114.29±6.64

DPPH 2,2-diphenyl-1-picrylhydrazyl radical scavenging activity, CUPRAC Copper reducing antioxidant capacity TE: Trolox Equivalent; dw: dry weight.
